# High Housing Density-Induced Chronic Stress Diminishes Ovarian Reserve via Granulosa Cell Apoptosis by Angiotensin II Overexpression in Mice

**DOI:** 10.3390/ijms23158614

**Published:** 2022-08-03

**Authors:** Jihyun Kim, Sooseong You

**Affiliations:** KM Convergence Research Division, Korea Institute of Oriental Medicine, Daejeon 34054, Korea; kimjihyun763@naver.com

**Keywords:** stress, transcriptomic changes, angiotensin II, granulosa cell, apoptosis, diminished ovarian reserve, mouse model

## Abstract

Repeated and prolonged stress causes hypothalamic-pituitary-adrenal (HPA) dysregulation. Excessive hypothalamic-pituitary-adrenal axis activity has been linked to inadequate activation of the hypothalamus-pituitary-ovarian axis, which controls the growth and development of ovarian follicles and oocytes. Therefore, we assessed the ovarian reserve under high-housing-density-induced prolonged stress, and investigated the mechanisms underlying diminished ovarian reserve in this study. Eight-week-old female C57BL/6 mice were housed for 10 weeks under different housing densities. We then assessed hormone levels, performed histology and immunohistochemistry analyses of ovarian follicles, evaluated ovarian mRNA expression, and measured angiotensin II-mediated apoptosis in vitro. More densely housed mice presented increased corticosterone levels and decreased follicle-stimulating and luteinizing hormone levels. Moreover, mice exposed to prolonged ordinary stress showed a reduced level of serum anti-Müllerian hormone and an increased number of atretic ovarian follicles. Stressed mice showed increased levels of angiotensinogen and angiotensin II in the ovaries and serum. Furthermore, our in vitro study confirmed that high-housing-density-related stress induced granulosa cell apoptosis, resulting in diminished ovarian reserves. Collectively, our findings highlight the importance of women managing everyday stress to maintain their reproductive health.

## 1. Introduction

With the changes in society, culture, and living environment, women are more likely to suffer from poor psychophysiological health [1]. Repeated and prolonged stress induces hypothalamic-pituitary-adrenal (HPA) dysregulation, which disrupts homeostasis [2]. Activation of the HPA axis results in the secretion of various stress hormones including glucocorticoids, corticotropin-releasing factor (CRF), and cortisol [3]. Furthermore, excessive HPA activity is reportedly associated with inadequate activation of the hypothalamus-pituitary-ovarian axis, which controls the growth and development of ovarian follicles and oocytes [4].

The ovary is the most dynamic organ in mammals. Follicular degeneration or atresia can occur at any stage of folliculogenesis during the estrous cycle to maintain ovarian homeostasis [5]. Physiological stress negatively correlates with the serum anti-Müllerian hormone (AMH) level, a marker for reproductive potential [6]. A low AMH level accelerates primordial follicle pool depletion, which can further reduce fertility [7]. The generally accepted mechanism is that cortisol levels, elevated by various stressors, interrupt steroid biosynthesis and gonadotropin release maintenance to induce follicular atresia [8,9]. These impaired conditions can cause irregular estrous cycles and anovulation in stressed women and mice [10]. Overall, stress exposure exerts a negative effect on reproductive potential by decreasing the ovarian reserve.

To elucidate the underlying etiology of stress disorders, researchers have used animal models of stress induced by a variety of acute unpredictable stresses, including behavior restriction, fasting, water deprivation, forced swimming, and tail and foot shock [11,12]. However, these stressors can lead to weight loss, with negative effects on the body. These sources of stress are not designed to mimic everyday human stress, such as a stressful job and familial stress.

Population density has been suggested to affect social interactions, and the overcrowding of mice has been shown to increase social interactions and stress hormones [13]. Indeed, male C57BL/6 mice housed in a restricted space have been shown to experience more stress and present high corticosterone levels [14]. A previous study indicated that dense housing conditions over a short period did not cause stress in female mice [15]. However, there has been no report on whether chronic stress induced by increased population density affects ovarian reserve. Thus, in this study, we assessed the ovarian reserve in mice under prolonged daily stress caused by overcrowding, and investigated the underlying biological mechanisms of diminished ovarian reserve.

## 2. Results

### 2.1. High Housing Density Induces Physiological Stress without Weight Loss

**Figure 1:**
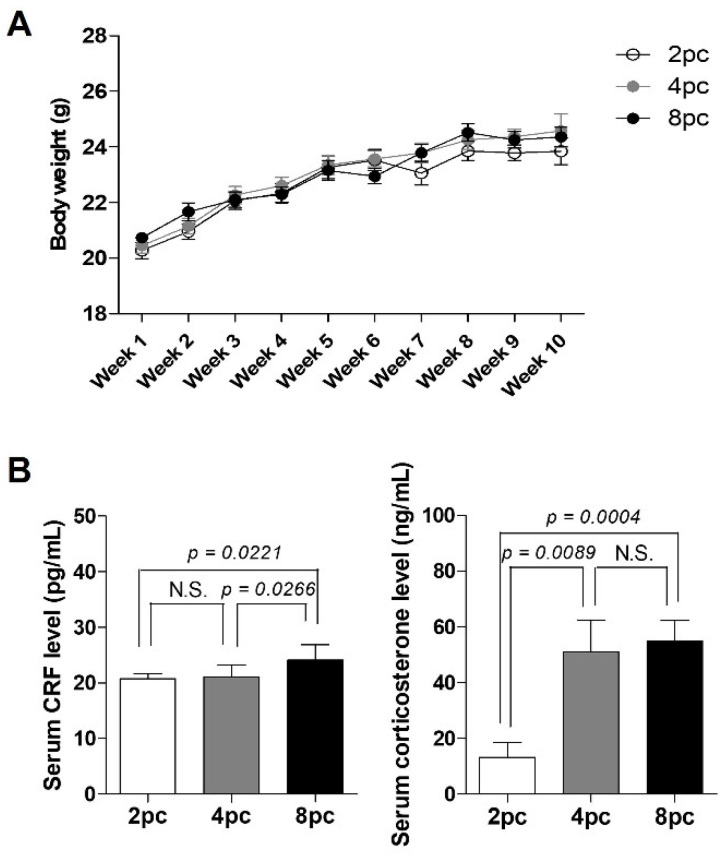
Body weight changes and stress hormone levels in mice housed at different densities for 10 weeks. (**A**) Body weight changes (*n* = 8 for each group). (**B**) Serum levels of corticotropin-releasing factor (CRF) and corticosterone (*n* = 8 for each group). Data are presented as mean ± standard error of mean (SEM). Statistical analyses were performed using Student’s *t*-test. 2pc, mice housed at two mice per cage (white bar); 4pc, mice housed at four mice per cage (gray bar); 8pc, mice housed at eight mice per cage (black bar); N.S., not significant.

The mice were housed at two (2pc), four (4pc), or eight (8pc) per cage for 10 weeks and were monitored weekly throughout the study. The body weights of all mice gradually increased over the 10-week housing period; however, the difference was not significant (Figure 1A). We measured serum stress hormone levels among the groups to assess stress levels. Corticosterone levels significantly increased in the 4pc and 8pc mice compared with those in the 2pc mice (*p* < 0.05; Figure 1B), indicating that the more densely housed mice were chronically stressed without body weight loss via regulation of the HPA axis activity.

### 2.2. High Housing Density Induces Hormonal Imbalance

**Figure 2:**
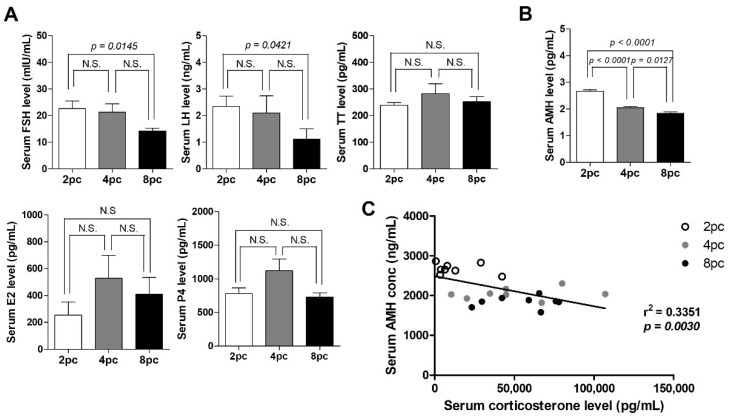
Serum hormone levels in mice housed at different densities for 10 weeks. (**A**) Serum follicle-stimulating hormone (FSH), luteinizing hormone (LH), estradiol (E2), progesterone (P4) and total testosterone (TT) levels. (**B**) Levels of serum anti-Müllerian hormone (AMH). (**C**) Correlation between the serum AMH and corticosterone levels (*n* = 8 for each group). Data are presented as mean ± SEM. Statistical analyses were performed using Student’s *t*-test and linear regression. 2pc, mice housed at two mice per cage (white bar); 4pc, mice housed at four mice per cage (gray bar); 8pc, mice housed at eight mice per cage (black bar); N.S., not significant.

To investigate the effect of housing density on the hypothalamus-pituitary-ovarian axis activity, we measured the serum levels of steroid hormones including follicle-stimulating hormone (FSH), luteinizing hormone (LH), estradiol (E2), progesterone (P4) and total testosterone (TT). FSH and LH levels significantly decreased in the 8pc mice compared with those in the 2pc mice (*p* < 0.05; Figure 2A), whereas E2, P4 and TT levels were comparable between the groups (Figure 2A). Ovarian reserve was assessed by measuring serum AMH level, which was significantly lower in the 4pc and 8pc mice than in the 2pc mice (*p* < 0.05; Figure 2B). We also found a negative correlation between the serum AMH and corticosterone levels (*p* < 0.05; Figure 2C).

### 2.3. High Housing Density Induces Follicle Loss

**Figure 3:**
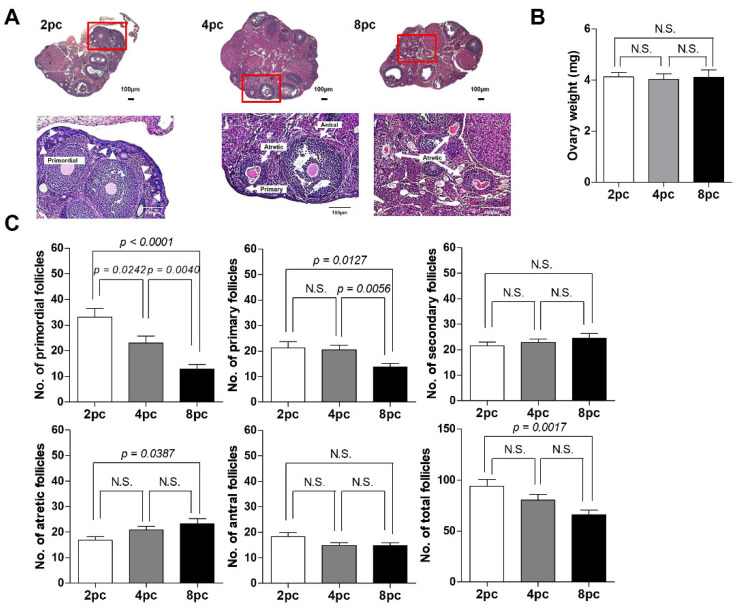
Histological analysis of the ovarian follicles in mice housed at different densities for 10 weeks. (**A**) After housing for 10 weeks, mouse ovaries were assessed histologically (*n* = 8 for each group). (**B**) Ovary weight. (**C**) Number of ovarian follicles at different stages and total number of ovarian follicles. Data are presented as mean ± SEM. Statistical analyses were performed using Student’s *t*-tests. 2pc, mice housed at two mice per cage (white bar); 4pc, mice housed at four mice per cage (gray bar); 8pc, mice housed at eight mice per cage (black bar); N.S., not significant.

Next, the ovaries from mice in each group were histologically observed (Figure 3A). The ovary weight did not differ among the three groups (Figure 3B). Compared with the 2pc mice, high-density housing conditions lowered the number of primordial follicles (*p* < 0.05; Figure 3C). The number of primary follicles was significantly reduced in the 8pc mice compared with that in the 2pc and 4pc mice. However, there were no differences in the number of growing follicles among the 2pc, 4pc, and 8pc mice (Figure 3C). Interestingly, the number of atretic follicles increased significantly in the 8pc mice compared with that in the 2pc mice (Figure 3C).

### 2.4. High Housing Density Induces Changes in Ovarian mRNA Expression

**Figure 4:**
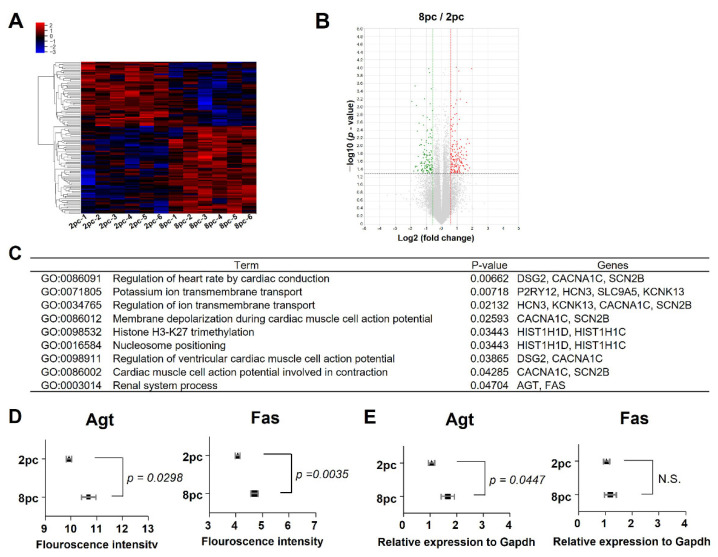
Hierarchical clustering and analysis of differentially expressed mRNAs. RNA sequencing was performed to compare gene expression in the ovaries of mice (2 or 8 mice per cage) housed for 10 weeks. (**A**) Hierarchical clustering among the mRNA expression profiles (*n* = 6 for each group) and (**B**) volcano plot revealing 116 differentially expressed genes (DEGs) with fold-changes > 1.5 and *p* < 0.05. Red and green dots indicate up and downregulated differentially expressed mRNAs, respectively. (**C**) Differentially expressed mRNAs were subsequently classified based on their enrichment in biological processes. (**D**) Sequencing fluorescence intensities indicate the expression levels of angiotensinogen (Agt) and Fas cell surface death receptor (Fas). (**E**) Reverse transcription quantitative polymerase chain reaction (RT-qPCR) was performed to verify the differential expression of Agt and Fas. Data are presented as mean ± SEM. Statistical analyses were performed using Student’s *t*-test. 2pc, mice housed at two mice per cage (triangle); 8pc, mice housed at eight mice per cage (square); N.S., not significant.

We performed QuantSeq 3′ mRNA sequencing to compare mRNA expression patterns in the ovaries of the 2pc and 8pc mice. Among the 13,668 genes that were identified, hierarchical clustering analysis and volcano plot showed 116 differentially expressed genes (DEGs), with a fold-change of >1.5 between the two mouse groups (*p* < 0.05, Figure 4A,B). Of these, 67 (57.8%) were upregulated and 49 (42.2%) were downregulated in the 8pc mice compared with those in the 2pc mice (Supplementary Tables S1 and S2). The Gene Ontology (GO) analysis of 2pc and 8pc mouse datasets revealed that the DEGs were enriched in biological processes, such as ion transmembrane transport, histone trimethylation, and renal system process (Figure 4C). The ovarian renin–angiotensin system reportedly plays a role in ovarian physiology [16], and two genes involved in the GO term renal system process, angiotensinogen (Agt) and Fas cell surface death receptor (Fas), were differentially expressed between the groups (Figure 4D). Both genes were then validated using reverse transcription quantitative polymerase chain reaction (RT-qPCR) with a fluorescent probe-based TaqMan assay (Figure 4E). The ovarian Agt expression was significantly higher in the 8pc mice than in the 2pc mice. However, the RNA sequencing (RNA-Seq) and RT-qPCR data of Fas were not consistent, likely due to the RNA-Seq filtering conditions for measuring quality, including fold-change > 1.5 and *p* < 0.05 [17].

### 2.5. Housing Density Induces Changes in the Ovarian Renin-Angiotensin System

**Figure 5:**
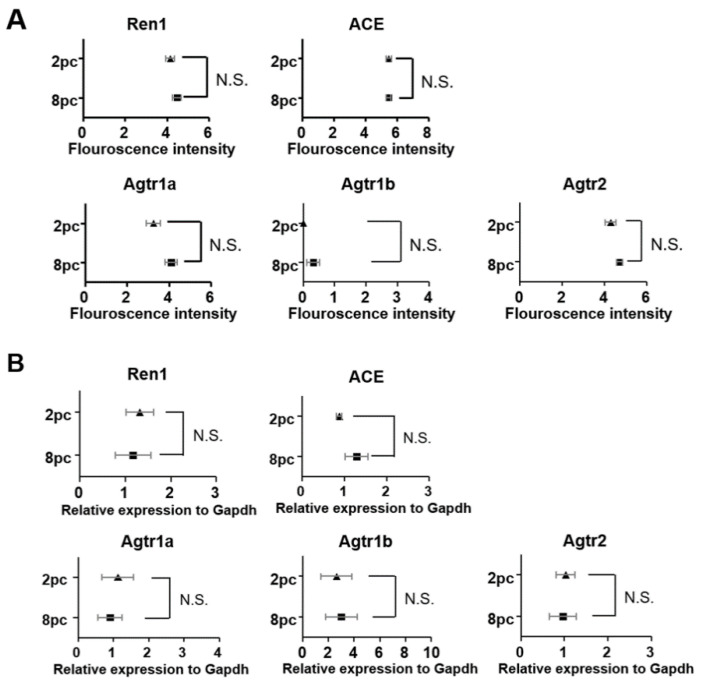
Expression of genes involved in the ovarian renin-angiotensin system. (**A**) Sequencing fluorescence intensities indicate the expression levels of Ren1, Ace, Agtr1a, Agtr1b, and Agtr2. (**B**) RT-qPCR was performed to verify the differential expression of these five genes. Data are presented as mean ± SEM. Statistical analyses were performed using Student’s *t*-test. 2pc, mice housed at two mice per cage (triangle); 8pc, mice housed at eight mice per cage (square); N.S., not significant.

Next, we analyzed gene expression associated with the ovarian renin-angiotensin system, including renin 1 (Ren1), angiotensin-converting enzyme (Ace), angiotensin II receptor type 1a (Agtr1a), angiotensin II receptor type 1b (Agtr1b), and angiotensin II receptor type 2 (Agtr2). However, the expressions of these five genes were comparable between the 2pc and 8pc mice (Figure 5A), which was verified using RT-qPCR with TaqMan assay (Figure 5B).

**Figure 6:**
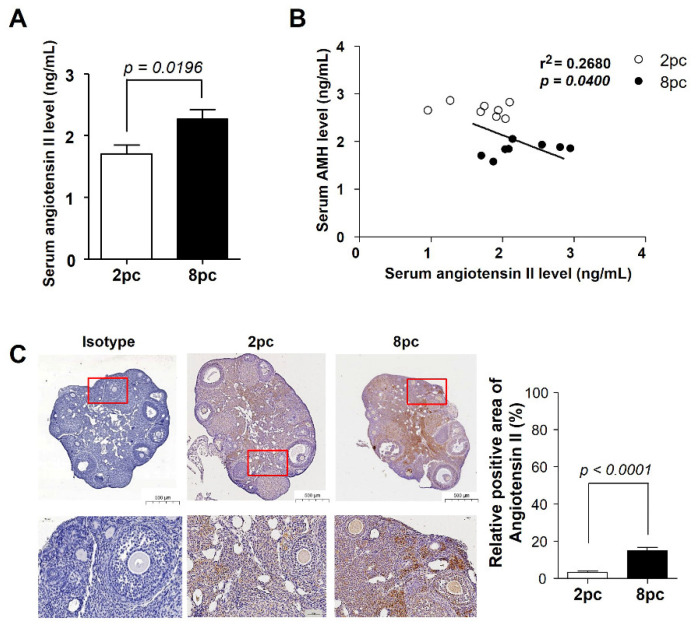
Serum angiotensin II levels and immunohistochemical examination of angiotensin II in the ovaries. (**A**) Serum angiotensin II level (*n* = 8 for each group). (**B**) Correlation between serum AMH and angiotensin II levels. (**C**) Immunohistochemistry of angiotensin II in the ovaries. Data are presented as mean ± SEM. Statistical analyses were performed using Student’s *t*-test. 2pc, mice housed at two mice per cage (white bar); 8pc, mice housed at eight mice per cage (black bar).

We then measured the level of angiotensin II in the serum and ovaries. The serum level of angiotensin II was significantly higher in the 8pc mice than in the 2pc mice (*p* < 0.05; Figure 6A). We also observed a negative correlation between the serum AMH and angiotensin II levels (Figure 6B). In addition, the results of immunohistochemistry revealed that angiotensin II was expressed in the ovarian granulosa cells and stroma (Figure 6C). Angiotensin II expression was significantly higher in the ovaries of the 8pc mice than in those of the 2pc mice, indicating that local ovarian angiotensin II expression was upregulated by chronic-stress-induced Agt expression, which may be associated with diminished ovarian reserves.

### 2.6. Angiotensin II Induces Granulosa Cell Apoptosis In Vitro

**Figure 7:**
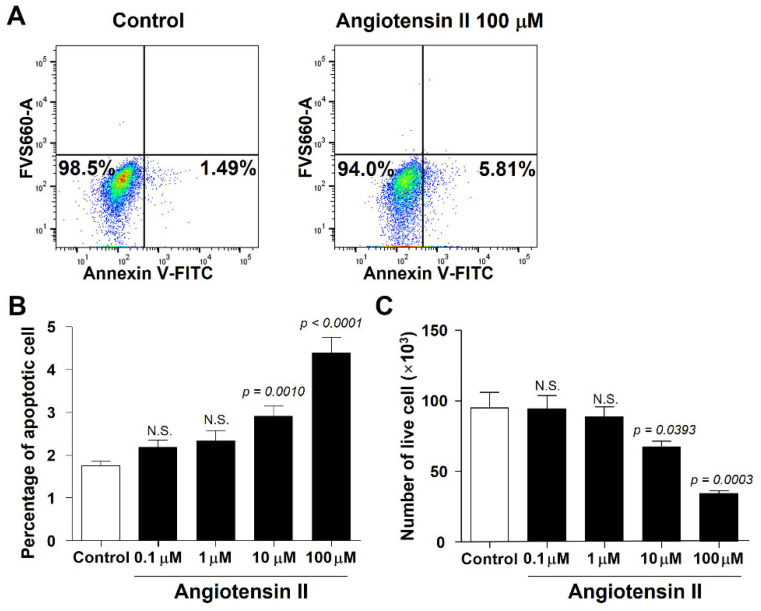
Viability of a human granulosa cell line after angiotensin II treatment. (**A**) Flow cytometry was performed to discriminate live cells from dead and apoptotic hGL5 granulosa cells after angiotensin II treatment. (**B**) Percentage of apoptotic cells (*n* = 6 for each group). (**C**) Number of live cells (*n* = 6 for each group). White and black bars indicate non-treated and angiotensin II-treated group, respectively. Data are presented as mean ± SEM. Statistical analyses were performed using Student’s *t*-test. N.S., not significant.

To investigate the role of angiotensin II in the ovarian reserve, the viability of human granulosa cells was analyzed using flow cytometry after treatment with angiotensin II (Figure 7A). The percentage of apoptotic cells significantly increased after treatment with 10 and 100 μM angiotensin II, whereas the number of live cells significantly decreased (*p* < 0.05; Figure 7B,C). These results indicate that increased angiotensin II expression due to high-housing-density-induced chronic stress causes granulosa cell apoptosis, resulting in diminished ovarian reserves.

## 3. Discussion

Social overcrowding is an unintended environmental stressor that influences human health and behavior [18]. People exposed to high-density living conditions experience unwanted social interaction, which is associated with poor psychophysiological health [19]. Mouse housing density, an environmental variable in biomedical research, can induce ordinary stress that mimics human experiences, alters psychophysiology, and subsequently affects reproductive potential [20]. Mice exposed to intense stressors, such as behavior restriction and water deprivation, display a steady and rapid reduction in body weight [21], which is the earliest sign of imminent death [22]. Our study suggests that prolonged high-density housing adequately simulates ordinary stress without body weight changes. In addition, we provide evidence of the effect of high-housing-density stress on ovarian reserve.

High housing density induces steroid hormonal dysregulation that negatively affects the physiology of the mammalian ovary. Abnormally low FSH levels with low LH levels indicate a problem in the pituitary gland or hypothalamus functioning, which plays a vital role in ovulation [23]. AMH modulates primordial follicular growth initiation in the cluster of growing follicles to maintain the female reproductive life span [24]. Meanwhile, stress results in low AMH levels that lead to faster exhaustion of primordial follicles, despite lower FSH levels. As a consequence, high levels of stress hormone and angiotensin II have been implicated in triggering granulosa cell death, resulting in follicular atresia. Indeed, the total number of follicles significantly decreased in the 8pc mice compared with that in the 2pc and 4pc mice in the present study. Disruption of this hormonal balance causes follicle depletion and subsequent infertility. Interestingly, as ordinary housing conditions could affect the ovarian condition, leading to a low AMH level and primordial follicles, as observed in the 4pc mice, researchers should consider determining the effect of mouse caging density in animal studies on female reproductive biology.

High-housing-density-induced ordinary stress increases glucocorticoid and stress hormone levels. A prolonged increase in cortisol levels impairs follicular growth and development, and deteriorates oocyte quality [25]. Glucocorticoids increase AGT levels, influencing ovulatory capacity, supporting an important role of the ovarian renin–angiotensin system in ovarian function. We present evidence that mice stressed by high housing density display an upregulated AGT expression. More densely housed mice showed increased serum and local ovarian angiotensin II levels; furthermore, increased angiotensin II levels induced granulosa cell apoptosis in vivo. In rodents, treatment with angiotensin II has been shown to modulate ovarian steroidogenesis in vitro within cultured granulosa and theca cells, causing atresia [26]. In addition, apoptotic granulosa cells show an increase in the expression of ATGR2 and AGTR2 [27,28]. Dysregulation of the ovarian renin–angiotensin system may induce follicular atresia [29]. The negative correlation between serum AMH and angiotensin II levels could enable the development of new therapeutic approaches for women with diminished ovarian reserve.

Angiotensinogen is also involved in oocyte ovulation from selected preovulatory follicles under the control of FSH, via the regulation of plasminogen activator/plasminogen activator inhibitor in rodent ovaries [30,31]. Poor ovarian responders with increased AGTR2 expression show decreased oocyte maturation and quantity [32]. An abnormal ovarian renin–angiotensin system has been associated with ovarian pathologies such as polycystic ovarian syndrome, ovarian hyperstimulation syndrome, and ovarian cancer [33]. Therefore, it is necessary to investigate whether the stress induced by high housing density affects oocyte maturation, ovulatory processes, and ovarian pathogenesis.

Although the morphology and gene expression of ovaries are affected by the estrus stage, we did not evaluate the estrus stage. This is because the analysis itself could act as a stressor, exerting effects greater than those of stress caused by overcrowding. However, as there were no differences in the serum E2 and P4 levels among the three groups, all three groups could be considered to have been in a similar estrus stage.

The results of chronic stress by high housing density could be used for various research topics. First, it can be predicted that chronic stress also affects the cardiovascular system. In fact, many genes related to the cardiovascular system were derived through GO analysis (Figure 4C). Second, whether such chronic stress could be reduced by providing a private physical space is a very interesting topic. For example, we can put paper tubes in a cage and assess the level of stress after 10 weeks. Third, this chronic stress model could be used to develop foods or drugs that could prevent chronic stress.

## 4. Materials and Methods

### 4.1. Mice

All experiments and analyses were conducted in accordance with relevant guidelines and regulations. The study was also performed in compliance with the ARRIVE guidelines. Female C57BL/6 mice aged 8 weeks (OrientBio, Seongnam, Korea) were housed under specific pathogen-free conditions in individually vented cages (Optimice, Animal Care System, Centennial, CO, USA), each with a floor area of 484 cm^2^ for five 25 g mice. The mice were randomly assigned to a housing density of two, four, or eight mice per cage and housed for 10 weeks. The mice were subsequently euthanized with 1.2% avertin (0.6 mL/mouse; Sigma-Aldrich, St. Louis, MO, USA). Blood samples were collected, and sera were separated and stored at −80 °C until further analysis. The ovaries were removed and immediately placed in 4% paraformaldehyde (Biosesang, Seongnam, Korea) and liquid nitrogen for histological observation and RNA-Seq, respectively.

### 4.2. Enzyme-Linked Immunosorbent Assay for Hormone Assessment

The serum concentrations of CRF, corticosterone, FSH, LH, E2, P4, TT, AMH, and angiotensin II were measured using hormone-specific enzyme-linked immunosorbent assay kits according to manufacturer’s instructions.

For CRF (Cusabio Biotech Co., Wuhan, China), both intra- and inter-assay coefficients of variation (CVs) were <15%, with a sensitivity of 0.675 pg/mL. For corticosterone (Enzo Life Sciences, Farmingdale, NY, USA), the inter- and intra-assay CVs were 13.1% and 8.4%, respectively, and the sensitivity was 26.99 pg/mL. For FSH (Cusabio Biotech Co.), both intra- and inter-assay CVs were <15%, with a sensitivity of 2.5 mIU/mL. For LH (Endocrine Technologies, Newark, CA, USA), the intra- and inter-assay CVs were 7% and 15%, respectively, and the functional sensitivity was 5.2 ng/mL. For E2 (Enzo Life Sciences), the inter- and intra-assay CVs were <7.4% and <9.2%, respectively, and the sensitivity was typically <28.5 pg/mL. For P4 (Cusabio Biotech Co.), both intra- and inter-assay CVs were <15%, with a sensitivity of 0.2 ng/mL. For TT (LSbio, Seattle, WA, USA), after steroid replacement agent treatment, intra- and inter-assay CVs were <10% and <12%, respectively, and the sensitivity was typically 1 pg/mL. For AMH (AnshLabs, Webster, TX, USA), the inter-assay CV was <10%, with a sensitivity of 0.06 ng/mL. For angiotensin II (Enzo Life Sciences), the inter- and intra-assay CVs were <7.3% and <15.9%, respectively, and the sensitivity was 4.5 pg/mL.

### 4.3. Histological Assessment of Ovarian Follicles

Ten weeks after housing the mice at different densities, both ovaries were serially sectioned to obtain 5-μm-thick tissue sections, which were stained with hematoxylin and eosin. Primordial, primary, secondary, preovulatory, and atretic follicles, with visible oocytes, were counted in every tenth stained section to avoid counting the same follicle twice. The follicular stages were classified as previously described [34]: primordial follicles, with a single flat layer of granulosa cells surrounding the oocyte; primary follicles, with a single cuboidal granulosa cell layer; secondary follicles, with at least two granulosa cell layers and a theca layer; preovulatory follicles, with a complete antrum and theca layer; and atretic follicles, with a big antrum filled with follicular fluid and abnormal oocytes surrounded by cumulus cells.

### 4.4. RNA-Seq for mRNA Expression and Functional Annotation Analysis

Total RNA was extracted from ovary tissue collected from mice after 10 weeks of housing using TRIzol reagent (Invitrogen, Carlsbad, CA, USA) according to the manufacturer’s instructions. The purity and integrity of the extracted RNA was evaluated using NanoDrop ND-1000 UV-Vis spectrophotometer (Thermo Fisher Scientific, Waltham, MA, USA). All samples showed high purity (optical density 260/280 ratio > 1.80) and integrity (RNA integrity number > 7.0). Fold-changes of >1.5 and *p* < 0.05 were used as thresholds to identify DEGs. Gene enrichment and functional annotation analyses of the DEGs were performed using GO (accessed on 2 February 2022, http://geneontology.org) to identify potential biological functions of relevance. RNA-Seq data generated in this study were deposited at the National Center for Biotechnology Information (accessed on 8 December 2021, https://www.ncbi.nlm.nih.gov/sra/PRJNA787028).

### 4.5. Validation of Selected DEGs in the Ovaries

Significantly different genes of interest were validated using RT-qPCR to confirm the mRNA sequencing results. Complementary DNAs (cDNAs) were synthesized from the extracted total RNA using the iScript cDNA Synthesis Kit (Bio-Rad Laboratories, Hercules, CA, USA) according to the manufacturer’s instructions. qPCR was performed with a final reaction mixture volume of 20 µL, using the QuantStudio 6 Flex Real-time PCR system with TaqMan fluorescent probe-based detection, according to the manufacturer’s instructions (Thermo Fisher Scientific). The cycle threshold was normalized and compared using Gapdh as the internal control (Thermo Fisher Scientific).

### 4.6. Immunohistochemical Observation of the Ovaries

The paraffin-embedded ovarian tissues were sectioned to obtain 5-μm-thick tissue sections. The sections were deparaffinized in xylene. Endogenous peroxidases were inactivated with 3% hydrogen peroxide, followed by antigen retrieval in citrate buffer for 10 min at 97 °C. After blocking nonspecific binding using 1% bovine serum albumin (Sigma-Aldrich), angiotensin II immunostaining was performed using horseradish peroxidase (HRP)-conjugated anti-angiotensin II polyclonal IgG antibody (LSBio, Seattle, WA, USA), at a working dilution of 1:100 for 1 h at 3 °C. As the negative control, an HRP-conjugated isotype-matched IgG antibody (Abcam, Waltham MA, USA) was used. The angiotensin II staining area was calculated using ImageJ software version 1.53r 21 (National Institutes of Health, Bethesda, MD, USA), as described previously [35].

### 4.7. Culture of Human Granulosa Cell Line

The human granulosa cell line (hGL5) was purchased from Applied Biological Materials (Richmond, BC, Canada) and cultured in Prigrow IV medium (Applied Biological Materials), supplemented with 1× Insulin-Transferrin-Selenium (ITS; Zen-Bio, Durham, NC, USA), 4% Ultroser G serum substitute (Pall Corporation, Port Washington, NY, USA), 2% calf serum (Hyclone, Logan, UT, USA), and 1% penicillin/streptomycin (P/S; Gibco), according to the manufacturers’ instructions. The hGL5 cells (2 × 10^4^/well) were seeded in six-well collagen-coated plates (Corning, NY, USA) for 24 h and treated with 0–100 µM angiotensin II (Sigma-Aldrich) for 24 h in a low-serum-culture medium containing 1× ITS, 0.4% Ultroser G serum substitute, and 1% P/S. The cells were then collected from the culture medium for flow cytometry analysis.

### 4.8. Flow Cytometry of the Human Granulosa Cell Line

To differentiate live cells from dead and apoptotic cells, the hGL5 cells were stained with 1:10,000 BD Horizon Fixable Viability Stain 660 (BD Biosciences, San Jose, CA, USA) and 5 μL Annexin V-phycoerythrin antibody per tube (BioLegend, San Diego, CA, USA) for 15 min in a modified staining buffer containing culture medium and Annexin V buffer (BioLegend) at a 1:1 ratio on ice. The labeled cells were washed with modified staining buffer and analyzed using flow cytometry on a BD LSRFortessa Cell Analyzer (BD Biosciences). Before flow cytometry, 50 μL of CountBright Absolute counting beads (Invitrogen) were mixed with the cell samples in a calibration tube, according to the manufacturer’s instructions (target cell count in a tube = number of target cells/number of counted beads in each sample × assigned bead count in the 50 μL of counting beads) [36].

### 4.9. Statistical Analysis

Data are presented as mean ± standard error of the mean and were compared using the independent (two-tailed) *t*-test in GraphPad Prism version 8.4.0 (GraphPad Software Inc., La Jolla, CA, USA). Correlation and linear regression analyses among AMH, corticosterone, and angiotensin II levels were performed. Significance was defined at *p* < 0.05.

## 5. Conclusions

Our study demonstrated that prolonged ordinary stress induced by high housing density increases stress hormones such as CRF and corticosterone without body weight loss, and diminishes primordial and primary follicles with an increase in atretic follicles. We confirmed an increase in the angiotensin II level in the ovarian tissue of 8pc mice using immunohistochemistry, and verified that angiotensin II induced human granulosa cell apoptosis in vitro. This stress model may be widely applied in studies on ordinary stress responses that influence ovarian steroidogenesis and ovarian function. Prolonged ordinary stress has potential implications in ovarian pathogenesis. Hence, to maintain reproductive health, women must actively manage daily stress.

## Data Availability

The RNA-Seq data have been deposited at the National Center for Biotechnology Information (accessed on 8 December 2021, https://www.ncbi.nlm.nih.gov/sra/PRJNA787028).

## Supplementary Materials

The following supporting information can be downloaded at: https://www.mdpi.com/article/10.3390/ijms23158614/s1.
