# Supplementary material for: High Housing Density-Induced Chronic Stress Diminishes Ovarian Reserve via Granulosa Cell Apoptosis by Angiotensin II Overexpression in Mice
Source: Int J Mol Sci. 2022 Aug 3;23(15):8614. doi: 10.3390/ijms23158614 (PMC9369192; doi:10.3390/ijms23158614)
Supplement: Supplementary file 1 [file ijms-23-08614-s001.zip › Supplementary Table S1.pdf]

Supplementary Table S1. List of genes upregulated in 8pc mice compared with their expression in 2pc mice

Gene symbol	Gene_ID	8pc vs 2pc.fc	P-value
Pcdhga11	protocadherin gamma subfamily A, 11	2.326	0.001
Pnoc	prepronociceptin	2.208	0.032
Mks1	Meckel syndrome, type 1	2.188	0.000
Snora30	small nucleolar RNA, H /ACA box 30	2.075	0.047
Bpifb1	BPI fold containing family B, member 1	2.067	0.009
Zfp677	zinc finger protein 677	2.052	0.012
1810008I18Rik	RIKEN cDNA 1810008I18 gene	2.040	0.003
Coro2a	coronin, actin binding protein 2A	2.031	0.023
Hist1h1c	histone cluster 1, H1c	1.988	0.001
C030013C21Rik	RIKEN cDNA C030013C21 gene	1.964	0.016
5031410I06Rik	RIKEN cDNA 5031410I06 gene	1.946	0.000
Cela2a	chymotrypsin-like elastase family, member 2A	1.940	0.046
Trim9	tripartite motif-containing 9	1.914	0.022
Skida1	SKI /DACH domain containing 1	1.903	0.018
Nat2	N-acetyltransferase 2 (arylamine N-acetyltransferase)	1.893	0.001
Gpr37	G protein-coupled receptor 37	1.882	0.033
Gm20199	predicted gene, 20199	1.858	0.014
Hist2h2ac	histone cluster 2, H2ac	1.852	0.004
Pgbd1	piggyBac transposable element derived 1	1.825	0.002
Agt	angiotensinogen (serpin peptidase inhibitor, clade A, member 8)	1.822	0.030
Dscc1	DNA replication and sister chromatid cohesion 1	1.783	0.040
Tmem72	transmembrane protein 72	1.771	0.035
Lrtm1	leucine-rich repeats and transmembrane domains 1	1.758	0.023
Gm10220	predicted gene 10220	1.728	0.001
Hist1h1d	histone cluster 1, H1d	1.725	0.047
Tec	tec protein tyrosine kinase	1.723	0.009
Rnf17	ring finger protein 17	1.710	0.022
Speer4a	spermatogenesis associated glutamate (E)-rich protein 4A	1.707	0.002
Vstm2b	V-set and transmembrane domain containing 2B	1.693	0.018
Speer4b	spermatogenesis associated glutamate (E)-rich protein 4B	1.690	0.003
Eya1	EYA transcriptional coactivator and phosphatase 1	1.670	0.034
Bcl6	B cell leukemia /lymphoma 6	1.665	0.004
Gm4432	predicted gene 4432	1.653	0.010
Mansc4	MANSC domain containing 4	1.650	0.030

Sh3bgrl2	SH3 domain binding glutamic acid-rich protein like 2	1.646	0.013
Cacna1c	calcium channel, voltage-dependent, L type, alpha 1C subunit	1.646	0.009
Abca6	ATP-binding cassette, subfamily A (ABC1), member 6	1.640	0.027
Srd5a1	steroid 5 alpha-reductase 1	1.638	0.003
Gemin8	gem (nuclear organelle) associated protein 8	1.637	0.019
Trim23	tripartite motif-containing 23	1.633	0.012
Rgs22	regulator of G-protein signaling 22	1.626	0.019
Spic	Spi-C transcription factor (Spi-1 /PU.1 related)	1.625	0.042
Slc27a6	solute carrier family 27 (fatty acid transporter), member 6	1.615	0.022
Hist1h4d	histone cluster 1, H4d	1.612	0.006
Zfp931	zinc finger protein 931	1.603	0.010
Gm15446	predicted gene 15446	1.595	0.047
Frrs1	ferric-chelate reductase 1	1.594	0.033
Zfp85	zinc finger protein 85	1.582	0.026
1810010H24Rik	RIKEN cDNA 1810010H24 gene	1.579	0.028
Cln5	ceroid-lipofuscinosis, neuronal 5	1.577	0.017
P2ry12	purinergic receptor P2Y, G-protein coupled 12	1.576	0.039
Fam72a	family with sequence similarity 72, member A	1.571	0.048
Gm10052	predicted pseudogene 10052	1.567	0.016
Fas	Fas (TNF receptor superfamily member 6)	1.567	0.004
Megf9	multiple EGF-like-domains 9	1.564	0.002
Gm15408	predicted gene 15408	1.562	0.041
Fbxo27	F-box protein 27	1.557	0.030
4930426D05Rik	RIKEN cDNA 4930426D05 gene	1.553	0.020
1700018B24Rik	RIKEN cDNA 1700018B24 gene	1.548	0.048
1700049G17Rik	RIKEN cDNA 1700049G17 gene	1.544	0.001
Slc12a5	solute carrier family 12, member 5	1.540	0.003
Zfp605	zinc finger protein 605	1.539	0.012
Mtap7d3	MAP7 domain containing 3	1.533	0.000
Tgds	TDP-glucose 4,6-dehydratase	1.531	0.004
Prcp	prolylcarboxypeptidase (angiotensinase C)	1.526	0.044
1700110K17Rik	RIKEN cDNA 1700110K17 gene	1.522	0.049
Wscd2	WSC domain containing 2	1.514	0.016

Comparison of 2pc and 8pc mouse data revealed 67 upregulated genes in the latter group with fold-changes > 1.5 and $P < 0.05$. 2pc, mice housed at two mice per cage; 8pc, mice housed at eight mice per cage.
