# Supplementary material for: High Housing Density-Induced Chronic Stress Diminishes Ovarian Reserve via Granulosa Cell Apoptosis by Angiotensin II Overexpression in Mice
Source: Int J Mol Sci. 2022 Aug 3;23(15):8614. doi: 10.3390/ijms23158614 (PMC9369192; doi:10.3390/ijms23158614)
Supplement: Supplementary file 1 [file ijms-23-08614-s001.zip › Supplementary Table S2.pdf]

Supplementary Table S2. List of genes downregulated in 8pc mice compared with their expression in 2pc mice

Gene symbol	Gene_ID	8pc vs 2pc.fc	P-value
Ccdc74a	coiled-coil domain containing 74A	0.666	0.035
Prr15	proline rich 15	0.666	0.040
Zfp287	zinc finger protein 287	0.665	0.001
2900008C10 Rik	RIKEN cDNA 2900008C10 gene	0.663	0.020
Entpd6	ectonucleoside triphosphate diphosphohydrolase 6	0.660	0.048
Fry	FRY microtubule-binding protein	0.658	0.032
P4ha3	procollagen-proline, 2-oxoglutarate 4-dioxygenase (proline 4-hydroxylase), alpha polypeptide III	0.655	0.040
Il3ra	interleukin 3 receptor, alpha chain	0.651	0.006
Prkcq	protein kinase C, theta	0.650	0.025
Camk2n2	calcium /calmodulin-dependent protein kinase II inhibitor 2	0.649	0.020
Scn2b	sodium channel, voltage-gated, type II, beta	0.645	0.029
Dsg2	desmoglein 2	0.644	0.004
4930562C15 Rik	RIKEN cDNA 4930562C15 gene	0.642	0.022
Dennd6b	DENN /MADD domain containing 6B	0.640	0.014
Slc9a5	solute carrier family 9 (sodium /hydrogen exchanger), member 5	0.638	0.036
4933404O12 Rik	RIKEN cDNA 4933404O12 gene	0.637	0.041
Rims2	regulating synaptic membrane exocytosis 2	0.636	0.004
Jsrp1	junctional sarcoplasmic reticulum protein 1	0.633	0.000
Cmtm8	CKLF-like MARVEL transmembrane domain containing 8	0.622	0.013
Wfdc1	WAP four-disulfide core domain 1	0.621	0.009
Hyal1	hyaluronoglucosaminidase 1	0.615	0.037
Zfp791	zinc finger protein 791	0.611	0.017
Chst4	carbohydrate (chondroitin 6 /keratan) sulfotransferase 4	0.609	0.041
Tcte2	t-complex-associated testis expressed 2	0.604	0.001
Xntrpc	Xndc1-transient receptor potential cation channel, subfamily C, member 2 readthrough	0.602	0.005
Wdr86	WD repeat domain 86	0.601	0.041
Lgals7	lectin, galactose binding, soluble 7	0.600	0.017
Hcn3	hyperpolarization-activated, cyclic nucleotide-gated K+ 3	0.600	0.040
Ptprv	protein tyrosine phosphatase, receptor type, V	0.592	0.038
Asphd2	aspartate beta-hydroxylase domain containing 2	0.591	0.027
Twist2	twist basic helix-loop-helix transcription factor 2	0.588	0.002
Tesc	tescalcin	0.585	0.000

Kif1a	kinesin family member 1A	0.582	0.049
Fam217b	family with sequence similarity 217, member B	0.581	0.017
Trpm6	transient receptor potential cation channel, subfamily M, member 6	0.577	0.032
Kcnk13	potassium channel, subfamily K, member 13	0.568	0.006
Gm11985	predicted gene 11985	0.568	0.000
Wfikkn2	WAP, follistatin /kazal, immunoglobulin, Kunitz, and netrin domain containing 2	0.568	0.046
Foxo6	forkhead box O6	0.567	0.006
Pih1d2	PIH1 domain containing 2	0.555	0.009
Foxd2os	forkhead box D2, opposite strand	0.539	0.017
5430431A17 Rik	RIKEN cDNA 5430431A17 gene	0.532	0.004
Gpd1	glycerol-3-phosphate dehydrogenase 1 (soluble)	0.526	0.042
Cst6	cystatin E /M	0.515	0.022
Per2	period circadian clock 2	0.512	0.017
Fam219a	family with sequence similarity 219, member A	0.511	0.041
Tssk4	testis-specific serine kinase 4	0.490	0.050
H2-Q10	histocompatibility 2, Q region locus 10	0.480	0.031
Islr2	immunoglobulin superfamily containing leucine-rich repeat 2	0.479	0.033

Comparison of 2pc and 8pc mouse data revealed 49 downregulated genes in the latter group with fold-changes > 1.5 and $P < 0.05$. 2pc, mice housed at two mice per cage; 8pc, mice housed at eight mice per cage.
